# The Efficacy, Safety, and Tolerability of Levofloxacin Quadruple Therapy for *Helicobacter pylori* Eradication: A Randomized, Double-Blind Clinical Trial

**DOI:** 10.1155/2022/9794901

**Published:** 2022-12-06

**Authors:** Fariborz Mansour-Ghanaei, Behnam Masihipour, Mohammad Fathalipour, Soheil Hassanipour, Homayoon Sokhanvar, Alireza Mansour-Ghanaei, Mehrnaz Asgharnezhad, Farahnaz Joukar

**Affiliations:** ^1^Caspian Digestive Diseases Research Center, Guilan University of Medical Sciences, Rasht, Iran; ^2^Gastrointestinal and Liver Diseases Research Center, Guilan University of Medical Sciences, Rasht, Iran; ^3^GI Cancer Screening and Prevention Research Center, Guilan University of Medical Sciences, Rasht, Iran; ^4^Department of Pharmacology and Toxicology, Faculty of Pharmacy, Hormozgan University of Medical Sciences, Bandar Abbas, Iran

## Abstract

The incidence of microbial resistance is growing, and new rescue regimens are needed to treat *Helicobacter pylori* (*H. pylori*) infection. This study aimed to evaluate levofloxacin-based quadruple therapies' efficacy, safety, and tolerability in eradicating *H. pylori*. In a randomized, double-blind clinical trial, 220 patients with dyspepsia and *H. pylori* infection were randomly assigned to receive either bismuth subcitrate 240 mg, pantoprazole 20 mg, amoxicillin 1000 mg twice a day, and levofloxacin 500 mg daily for seven days (BPAL-7) or ten days (BPAL-10). The eradication of *H. pylori* was evaluated two months after the end of treatment, and adverse drug reactions (ADRs) were assessed during the intervention. According to intention-to-treat and per-protocol, the eradication rate was significantly lower in the BPAL-7 regimen at 49.1% (95% CI: 39.3–57.8) and 47.6% (95% CI: 39.7–58.4), respectively, compared to the BPAL-10 regimen at 62.7% (95% CI: 53.6–72.8) and 62.4% (95% CI: 55.1–72.8), respectively. The ADR incidence was not statistically significant between the groups of BPAL-7 (33.6%) and BPAL-10 (36.7%). Although the ADRs were negligible in both groups, these regimens could not be an ideal alternative therapy for *H. pylori* because of their low eradication rates compared to standard regimens. *Trial Registration*. The study was reviewed and approved by the Iranian Registry of Clinical Trials (IRCT201406141155N19).

## 1. Introduction

*Helicobacter pylori (H. pylori)* affects about 80% of the total population in developing countries and 20–50% in developed countries [1]. This infection, as a worldwide health problem, is associated with acute and chronic gastritis, peptic ulcer disease, and gastric cancer [2–4]. The eradication of *H. pylori* has a main role in the prevention and treatment of these complications [5, 6]. However, the major concerns with *H. pylori* treatment are low compliance of patients, adverse drug reactions (ADRs), and microbial resistance to medicines [7, 8].

The efficacy of present pharmacological treatments for *H. pylori* infection has been evaluated in different studies [9–15]. The co-administration of a proton-pump inhibitor (PPI) and two antibiotics (triple therapy) or the addition of bismuth salts to the triple therapy (bismuth-based quadruple therapy) for a period of 14 days is the most common regimens [16]. Among antibacterial agents, amoxicillin or metronidazole, along with clarithromycin, is the first-line treatment for the eradication of *H. pylori* [10, 17]. Nevertheless, microbial resistance is one of the challenging factors of treatment, which causes the low efficacy of these regimens. Hence, effective antibiotics should be selected according to the regional pattern of *H. pylori* resistance [18, 19].

The prevalence of *H. pylori* infection is relatively high among the Iranian population [20, 21]. Moreover, novel strains that resist clarithromycin and metronidazole are prevalent in some geographical parts of Iran [22, 23]. Therefore, evidence suggested new antibiotic regimens with higher efficacy (eradication rates of >80%), lower ADRs and cost, and acceptable patient compliance. The efficacy of levofloxacin as a new-generation quinolone in the treatment of *H. pylori* infection has been assessed in several studies, and levofloxacin-containing regimens have shown various eradication rates [24–30].

The current study aimed to evaluate the efficacy, safety, and tolerability of a seven- and ten-day period of bismuth subcitrate, pantoprazole, amoxicillin, and levofloxacin regimens in the eradication of *H. pylori* among patients referred to the Gastrointestinal Outpatient Clinic of Razi Hospital, Rasht, Iran.

## 2. Methods

### 2.1. Trial Design

This study was a phase III open-label, randomized, double-blind, controlled trial carried out between July 2015 and November 2015 at the Gastrointestinal and Liver Diseases Research Center (GLDRC), Rasht, Iran. All included participants were randomly divided into one of the therapeutic regimens and signed informed consent. The eradication rate of *H. pylori* was assessed two months after the end of the intervention, and the incidence of ADRs and patient compliance were assessed at the end of treatment.

### 2.2. Ethical Consideration

The protocol of the study was approved by the local ethical committee of Guilan University of Medical Sciences (Ethics Committee Code: 1930175713) and also performed according to the Declaration of Helsinki for human subjects. The study was reviewed and approved by the Iranian Registry of Clinical Trials (IRCT201406141155N19).

### 2.3. Patients

Patients complaining of dyspeptic symptoms referred to the Gastrointestinal Outpatient Clinic of Razi Hospital, Rasht, Iran, were enrolled. The main inclusion criteria were a positive rapid urease test and being 15 to 65 years old. Patients with coexisting serious illnesses, including liver cirrhosis, renal failure, heart failure, gastrointestinal malignancies, a history of seizure or hematologic diseases, an allergy to medications, and an previous incomplete treatment course, as well as pregnant or nursing mothers, were excluded.

### 2.4. Randomization and Intervention

A block randomization method was used in order to assign patients randomly into permuted treatment blocks and ensure equal numbers in each group of treatments. One of these groups received a seven-day period of bismuth subcitrate 240 mg, pantoprazole 20 mg, amoxicillin 1000 mg twice a day, and levofloxacin 500 mg daily (BPAL-7), and the other received a ten-day period of the same regimen (BPAL-10).

Patients were recommended to take bismuth subcitrate, pantoprazole, levofloxacin before meals and amoxicillin after meals at the scheduled times. It was also advised to avoid smoking, drinking alcoholic or caffeinated beverages, eating spicy foods, and taking nonsteroidal antiinflammatory drugs or medications containing a monoamine oxidize inhibitor.

Demographic criteria, including age, gender, cigarette smoking, and alcohol drinking, were recorded.

### 2.5. Evaluation of *H. pylori* Eradication

Two months after the end of the intervention, a Heliprobe 14C-Urea Breath Test (14C-UBT, Kibion AB, Uppsala, Sweden) with a 95% sensitivity and 100% specificity was performed to evaluate the *H. pylori* eradication. Treatment success was defined as the result of <50 disintegrations per minute (DPM). Taking the medicines was stopped during these two months to avoid interaction with the results of 14C-UBT.

### 2.6. Assessment of ADRs and Patient Compliance

All patients were informed about potential ADRs, including pyrosis, anorexia, nausea, vomiting, bitter taste, abdominal colic, epigastric pain, headache, and back pain. The ADRs were evaluated during the intervention using a 0–10 scoring system (mild: 0–3, the ADRs exist but are intolerable; moderate: 4–6, ADRs sometimes interfere with daily life activity; severe: 7–10, ADRs interfere continuously with daily life activities). Acceptable patient compliance was defined as the consumption of >80% of the prescribed medicines [31].

### 2.7. Statistical Analyses

The eradication rates of the two regimens were determined using intention-to-treat (ITT) and per-protocol (PP) analysis. Patients who declined to proceed with the treatment or those with poor compliance were excluded from PP analysis; however, all patients, including patients who used the treatments out of the protocols or dropouts, were included in ITT analysis. The eradication rate of *H. pylori*, odd ratios, and 95% confidence interval were evaluated for each regimen. Chi-square and *t*-tests were employed to compare qualitative and quantitative variables between the two treatment groups, respectively. All statistical analyses were performed using SPSS, version 18.0 software (SPSS Inc., Chicago, IL, United States). A *P* < 0.05 was considered statistically significant.

## 3. Results

### 3.1. Characteristics of the Patients

**Figure 1:**
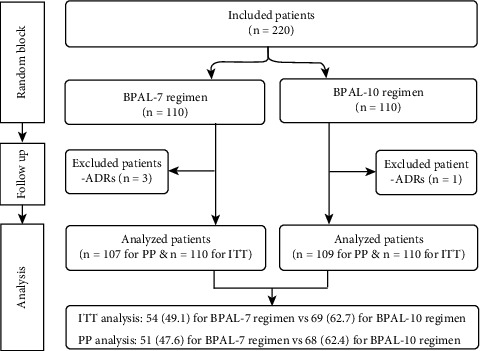
Flow chart for the process of patient randomization. ITT: intention-to-treat, PP: per-protocol, ADRs: adverse drug reactions, and BPAL-7 and BPAL-10: bismuth-based quadruple therapy containing pantoprazole, amoxicillin, and levofloxacin for seven and ten days, respectively.

A total of 220 patients with *H. pylori* infection were randomly assigned to either the BPAL-7 (*n* = 110) or BPAL-10 (*n* = 110) groups. Three patients in the BPAL-7 group (one female with severe epigastric pain and diarrhoea and two males with severe anorexia and back pain) and one patient in the BPAL-10 group (one female with severe epigastric pain and nausea) were excluded from the trial because of drug intolerance and low compliance. A flow diagram describing patient selection and study outcomes is shown in Figure 1.

No significant differences were observed between the two groups regarding age, gender, cigarette smoking, and alcohol drinking (*P* > 0.05). The basic demographic characteristics of the patients are demonstrated in Table 1.

### 3.2. Eradication Rates

The eradication rate of the BPAL-7 regimen (49.1%, 95% CI: 39.3–57.8) was lower than that of the BPAL-10 regimen (62.7%, 95% CI: 53.6–72.8) on ITT analysis (OR = 1.75, *P* < 0.05). In addition, PP analysis also demonstrated a lower eradication rate in the BPAL-7 regimen (47.6%, 95% CI: 39.7–58.4) than that of the BPAL-10 regimen (62.4%, 95% CI: 55.1–72.8) (OR = 0.54, *P* < 0.05). The eradication rates of the two regimens are represented in Table 2. Univariate analyses showed no association between age, gender, cigarette smoking, and alcohol drinking with an eradication rate of *H. pylori* in both groups (*P* > 0.05).

### 3.3. ADRs and Patient Compliances

Both BPAL-7 and PBAL-10 regimens were well tolerated by a majority of patients. The number of patients with ADRs was not statistically significant between the BPAL-7 group (36 patients, 33.6%) and the BPAL-10 group (40 patients, 36.7%). ADRs were rated as mild (55 patients, 25%), moderate (17 patients, 7.7%), and severe (4 patients, 1.8%). Both regimens' most-reported ADRs were epigastric pain, nausea, vomiting, and bitter taste (Table 3).

The compliance rates were 97.3% and 99.1% for the BPAL-7 and BPAL-10 regimens, respectively. Three patients in the BPAL-7 regimen and one in the BPAL-10 regimen were excluded from the PP analysis because they failed to ingest >80% of the medications.

## 4. Discussion

The present study's findings showed a lower rate of *H. pylori* eradication in the BPAL-7 regimen than in the BPAL-10 regimen among patients with dyspepsia and *H. pylori* infection. Both BPAL-7 and BPAL-10 regimens were well tolerated and had acceptable patient compliance.

Recent guidelines suggest the eradication rate of triple therapy with a PPI, amoxicillin, or metronidazole and clarithromycin as first-line regimens has decreased, particularly due to increased microbial resistance to clarithromycin [2]. Moreover, some recommended bismuth-based quadruple therapy regimens also have an eradication rate of less than 80% [32]. Therefore, more effective regimens are needed to eradicate the *H. pylori* infection.

Evidence suggests using levofloxacin, a new-generation fluoroquinolone with in vitro anti-*H. pylori* activity, as an alternative agent in clarithromycin-resistant cases. Levofloxacin-containing triple, quadruple, and sequential regimens have demonstrated various eradication rates [33–35].

A study compared a triple therapy consisting of esomeprazole, levofloxacin (500 mg daily), and amoxicillin with a standard regimen consisting of esomeprazole, metronidazole, bismuth, and tetracycline for 14 days. The eradication rate of the levofloxacin-containing regimen (96.3%) was higher than of the standard regimen (86.0%) in ITT analysis [25].

Gisbert et al. from Spain demonstrated the eradication rate of ranitidine bismuth citrate, levofloxacin, and amoxicillin as first-line triple therapy for *H. pylori* was 84.4, and ADRs, mainly including diarrhea, were reported in 9.5% of the patients [36]. This group performed a trial with 1000 patients on the efficacy of omeprazole, levofloxacin, and amoxicillin for ten days. They reported an eradication rate of 73.8% and ADRs of 20%, which most commonly included nausea, a metallic taste, and abdominal pain [26]. The levofloxacin dosage was higher in these studies (500 mg twice daily). In a recent multicenter study conducted by Gisbert et al., 14 days of treatment with esomeprazole, amoxicillin, levofloxacin (500 mg daily), and bismuth as a rescue therapy achieved more than 90% of the eradiation rate [27]. In another Spanish study, the eradication rate of omeprazole, levofloxacin (500 mg twice a day), and amoxicillin for ten days also had about a 20% failure rate [37].

A study from Kosovo randomized 105 patients to undergo either seven or ten days of levofloxacin-based regimens, including omeprazole, levofloxacin (500 mg daily), and amoxicillin. The eradication rates were 86.2% and 93.6%, respectively. About 5% of patients experienced ADRs, mainly nausea and diarrhea [28].

Several Taiwanese studies evaluated the efficacy of levofloxacin-containing regimens as rescue therapy for eradicating *H. pylori*. Kuo et al. reported the eradication rate of esomeprazole, amoxicillin, and levofloxacin (500 mg daily) regimen for seven days is similar to a second-linequadruple standard regimen (about 60–70%) [38]. Four years later, the same group published a study comparing levofloxacin-containing quadruple therapy (esomeprazole, bismuth, tetracycline, and 500 mg of levofloxacin once daily) for ten days with a high-dosemetronidazole-containing standard quadruple regimen. Based on their findings, eradication rates of about 80% were reached for both regimens [39]. On the other hand, Hsu et al. showed a ten-day quadruple therapy consisting of esomeprazole, bismuth, tetracycline, and levofloxacin (500 mg daily) achieved a very high eradication rate (95.8%) in this geographical area. ADRs were detected in 25.0% of patients [40]. In another study, the efficacy of adding bismuth to a levofloxacin-based triple regimen (rabeprazole, bismuth, amoxicillin, and 500 mg of levofloxacin daily) was assessed. The results revealed no further significant eradication rate (67.6%), but no more ADRs were noted [41].

According to the results of the present study, the BPAL-7 and PBAL-10 regimens were not clinically successful in the treatment of *H. pylori*, with an eradication rate of 62.7% and 49.1% by ITT analyses, respectively. Both regimens achieved neither the cut point of an ideal regimen (eradication rates of >90%) nor the acceptable eradication rates of Maastricht and other guidelines (>80%) [42]. The low efficacy of these regimens is likely related to the regional pattern of bacterial resistance. The results of these regimens are consistent with a previous Iranian study evaluating the effectiveness of a 14-day triple therapy comprising omeprazole, levofloxacin (500 mg daily), and amoxicillin with a success rate of 75% [43]. However, studies conducted in other parts of Iran revealed fluoroquinolone-containing regimens, including a 14-day of omeprazole, levofloxacin (250 mg twice a day), and amoxicillin, as well as a 14-day of omeprazole, levofloxacin (500 mg twice a day), and amoxicillin as rescue therapies for *H. pylori,* had eradication rates of 90% and 86.7%, respectively [44, 45]. Moreover, some other Iranian investigations evaluated the efficacy of levofloxacin-based, sequential therapy in treating *H. pylori*, and the eradication rate ranged from 70.8% to 85.1% [46–48].

No relationship was observed between patient demographic characteristics, including age, gender, cigarette smoking, and alcohol drinking, and the success rate of both the BPAL-7 and BPAL-10 regimens. Previous studies have also demonstrated the eradication rate of levofloxacin-containing regimens was not associated with age [45, 46, 48], gender [45–47], smoking status [25, 46], and education level [46].

Although high efficacy and compatibility with the pattern of regional bacterial resistance are the main properties of an ideal pharmacological regimen for eradicating *H. pylori*, tolerability, patient compliance, simplicity, and cost efficacy should not be neglected [31]. The compliance rates for the BPAL-7 and BPAL-10 regimens were 97.3% and 99.1%, respectively. Despite high compliance rates, ADRs were reported in one-third of patients. The most-reported ADRs were epigastric pain, nausea, vomiting, and bitter taste. Generally, BPAL-7 and BPAL-10 regimens were patient-compatible and well-tolerated.

### 4.1. Limitations

The main limitations of the present study are the lack of a regional pattern of antibiotic resistance and a local estimate of the *H. pylori* eradication rate, as well as the small number of patients in the groups. The absence of pretreatment susceptibility assessment for levofloxacin is another drawback. Moreover, the finding may not apply to patients with previous treatment failure and recurrence of the disease.

## 5. Conclusion

The BPAL-7 and BPAL-10 regimens were patient-compatible and well-tolerated. However, both regimens showed no satisfactory eradication rate. Therefore, these regimens could not be definitive alternative therapies for *H. pylori* eradication in this region. It is better to evaluate the efficacy and tolerability of this regimen in other geographical areas through more extended studies with a larger sample size.

## Figures and Tables

**Table 1:** Demographic characteristics of the study patients.

Characteristics	BPAL-7 regimen (*n* = 107)	BPAL-10 regimen (*n* = 109)	*P* value
Gender, male	39 (36.5)	40 (36.7)	0.888
Age	41.46 ± 13.25	44.16 ± 12.70	0.649
Cigarette smoking			
Yes	4 (3.7)	7 (6.4)	0.353
No	103 (96.3)	102 (93.6)	
Alcohol drinking			
Yes	1 (0.9)	3 (2.7)	0.313
No	106 (99.1)	106 (97.3)	

Values were demonstrated as numbers (percentages) and mean ± SD for categorical and continuous variables, respectively. Chi-square and *t*-tests were performed to compare categorical and continuous variables between groups, respectively. BPAL-7 and BPAL-10: bismuth-based quadruple therapies containing pantoprazole, amoxicillin, and levofloxacin for seven and ten days, respectively.

**Table 2:** The eradication rates of the BPAL-7 and BPAL-10 regimens.

Analyses	BPAL-7 regimen (*n* = 107)	BPAL-10 regimen (*n* = 109)	*P* value
Intention-to-treat	54 (49.1)	69 (62.7)	0.037
Per-protocol	51 (47.6)	68 (62.4)	0.024

Values were demonstrated as numbers (percentages). ^14^C-urea breath test was conducted to confirm the eradication of *H. pylori* two months after the end of treatment. A Chi-square test was performed to compare variables between groups. BPAL-7 and BPAL-10: bismuth-based quadruple therapies containing pantoprazole, amoxicillin, and levofloxacin for seven and ten days, respectively.

**Table 3:** The adverse drug reactions associated with the BPAL-7 and BPAL-10 regimens.

ADRs	BPAL-7 (*n* = 107)			BPAL-10 (*n* = 109)		
	Mild	Moderate	Severe	Mild	Moderate	Severe
Pyrosis	31 (29.0)	4 (3.7)	1 (0.9)	23 (21.1)	10 (9.2)	1 (0.9)
Anorexia	25 (23.4)	4 (3.7)	2 (1.9)	21 (19.3)	4 (3.7)	1 (0.9)
Nausea	19 (17.8)	2 (1.8)	1 (0.9)	25 (22.9)	0 (0.0)	0 (0.0)
Vomiting	2 (1.9)	1 (0.9)	0 (0.0)	0 (0.0)	0 (0.0)	1 (0.9)
Bitter taste	19 (17.8)	0 (0.0)	1 (0.9)	10 (9.2)	2 (1.8)	1 (0.9)
Abdominal colic	12 (11.2)	0 (0.0)	0 (0.0)	9 (8.2)	3 (2.7)	0 (0.0)
Epigastric pain	17 (15.9)	7 (6.5)	1 (0.9)	22 (20.2)	3 (2.7)	1 (0.9)
Headache	15 (14.0)	2 (1.9)	1 (0.9)	16 (14.7)	7 (6.4)	1 (0.9)
Back pain	6 (5.6)	1 (0.9)	2 (1.9)	9 (8.3)	6 (5.5)	1 (0.9)

Values were demonstrated as numbers (percentages). Adverse drug reactions were evaluated during the study. Chi-square test was performed to compare variables between groups. None of variables were shown statistically significant differences between two regimens in each category. ADRs: adverse drug reactions; BPAL-7 and BPAL-10: bismuth-based quadruple therapies containing pantoprazole, amoxicillin, and levofloxacin for seven and ten days, respectively.

## Data Availability

The datasets analyzed during the current study are available from the corresponding author on reasonable request.

## Abbreviations

*H. pylori*: *Helicobacter pylori*
ADRs: Adverse drug reactions
PPI: Proton-pump inhibitor
ITT: Intention-to-treat
PP: Per-protocol
BPAL-7: Bismuth-based quadruple therapy containing pantoprazole, amoxicillin, and levofloxacin for seven days
BPAL-10: Bismuth-based quadruple therapy containing pantoprazole, amoxicillin, and levofloxacin for ten days.
